# Draft Genome Sequence of an *Escherichia coli* Strain Isolated from a *Gallus gallus* Broiler Producing the Novel CTX-M-166 Variant

**DOI:** 10.1128/genomeA.01029-16

**Published:** 2016-10-06

**Authors:** Vera Manageiro, Lurdes Clemente, Sílvia Duarte, Luís Vieira, Manuela Caniça

**Affiliations:** a National Reference Laboratory of Antibiotic Resistances and Healthcare Associated Infections, National Institute of Health Doutor Ricardo Jorge, Lisbon, Portugal; b Centre for the Studies of Animal Science, Institute of Agrarian and Agri-Food Sciences and Technologies, Oporto University, Oporto, Portugal; c Instituto Nacional de Investigação Agrária e Veterinária (INIAV), Oeiras, Portugal; d Innovation and Technology Unit, Department of Human Genetics, National Institute of Health Doutor Ricardo Jorge, Lisbon, Portugal

## Abstract

We report here the draft genome sequence of the CTX-M-166-harboring O6:H16 sequence type 48 (ST48)-*fimH*34 *Escherichia coli* strain recovered from a *Gallus gallus* broiler. Sequence analyses revealed the presence of an IncI1/ST103-IS*Ecp1*-*bla*_CTX-M-166_-*orf477* plasmid region and of diverse antibiotic resistance and virulence-acquired genes.

## GENOME ANNOUNCEMENT

Animals are considered potential reservoirs of antimicrobial-resistant bacteria (1). In this study, we used whole-genome sequencing (WGS) to characterize the new CTX-M-166-harboring *Escherichia coli* strain recovered in May 2014 from a 6-week-old *Gallus gallus* broiler flock from an industrial poultry unit in the central region of Portugal, carrying a new amino acid substitution, compared with CTX-M-1.

Genomic DNA of *E. coli* LV13072 was extracted using DNeasy blood and tissue kit (Qiagen) and quantified using Qubit 1.0 fluorometer (Invitrogen). The Nextera XT DNA sample preparation kit (Illumina) was used to prepare sequencing libraries from 1 ng of genomic DNA, according to the manufacturer’s instructions. WGS was performed using 250-bp paired-end reads on a MiSeq (Illumina). Sequence reads were trimmed and filtered according to quality criteria and *de novo* assembled into contigs by means of CLC Genomics Workbench 8.5.1 (Qiagen), as previously described (2).

The *de novo*-assembled genome contains a total assembly length of 5,236,233 bp, with a mean coverage of about 225-fold; the G+C content was 49.3%. The analysis yielded 351 contigs, ranging from 402 bp to 197,210 bp, with a minimum of 12-fold coverage. Overall, the structural and functional annotation with NCBI Prokaryotic Annotation Pipeline (PGAP [http://www.ncbi.nlm.nih.gov/genome/annotation_prok/]) detected 4,982 coding sequences (CDSs), 13 noncoding RNAs (ncRNAs), four complete rRNAs, and 68 tRNAs.

*In silico* antimicrobial resistance analyses using ResFinder version 2.1 (3), with a threshold of 90% identity and a minimum length of 60%, revealed genes conferring resistance to β-lactams (*bla*_CTX-M-166_ [contig 249] and *bla*_TEM-1_ [contig 63]), aminoglycosides (*strA-strB* [contig 289]), tetracycline [*tet*(A)-type (contig 20)], sulfonamides (*sul2* [contig 289]), and trimethoprim (*dfrA14*-type [contig 257]). Seven virulence factors were also detected using VirulenceFinder version 1.5 (3): *iss* (contig 232), *gad* (contigs 29 and 125), *astA* (contig 123), *iroN* (contig 232), *iha* (contig 102), *mchF*-type (contig 57), *celb*-type (contig 46), and *cma*-type (contig 318) genes.

PlasmidFinder version 1.3 and pMLST version 1.4 tools (4) revealed the presence of sequence type 103 (ST103)-IncI1 and Col8282 plasmid types, with an identity of 100%.

The bioinformatics analysis of genetic relatedness (SerotypeFinder version 1.1 [5], MLST version 1.8 [6], and FimTyper version 1.0) assigned this isolate to O6:H16 ST48-*fimH*34. The total number of pathogenicity determinants present in the LV13072 genome, matching 564 pathogenic families, showed 93.2% certainty of the isolate being a human pathogen (7).

The *bla*_CTX-M-166_ gene differed from *bla*_CTX-M-1_ by one point mutation that leads to the amino acid substitution Ala120Val. It was found in a 4,218-bp contig, which was manually assembled overlapping contigs 249, 329, and 334, with a mean coverage of 36.4-fold and G+C content of 41.2%. An IS*Ecp1*-*bla*_CTX-M-166_-*orf477* region was found upstream of an IncI shufflon, interrupting the segment *shfB* of the site-specific recombination system (8). The closest match (94.7% query coverage and 100% identity) of the CTX-M-166-containing contig as identified by BLASTn analysis was the *E. coli* plasmid pIFM3804 (accession no. KF787110), a CTX-M-1 IncI1 plasmid found on a UK pig farm (9).

The information presented herein will enable further studies about the genetic background of *bla*_CTX-M-166_ and functional characterization of CTX-M-166 β-lactamase aiming to assess the potential impact of this new variant in veterinary settings, particularly under pressure caused by antibiotic exposure.

### Accession number(s).

This whole-genome shotgun project has been deposited at DDBJ/ENA/GenBank under the accession no. MCBU00000000. The version described in this paper is version MCBU01000000.

## References

[1] Caniça M, Manageiro V, Jones-Dias D, Clemente L, Gomes-Neves E, Poeta P, Dias E, Ferreira E 2015 Current perspectives on the dynamics of antibiotic resistance in different reservoirs. Res Microbiol 166:594–600. doi:10.1016/j.resmic.2015.07.009. 26247891

[2] Manageiro V, Sampaio DA, Pereira P, Rodrigues P, Vieira L, Palos C, Caniça M 2015 Draft genome sequence of the first NDM-1-producing *Providencia stuartii* strain isolated in Portugal. Genome Announc 3(5):e01077-15. doi:10.1128/genomeA.01077-15. 26404603 PMC4582579

[3] Kleinheinz KA, Joensen KG, Larsen MV 2014 Applying the ResFinder and VirulenceFinder Web services for easy identification of acquired antibiotic resistance and *E. coli* virulence genes in bacteriophage and prophage nucleotide sequences. Bacteriophage 4:e27943. doi:10.4161/bact.27943. 24575358 PMC3926868

[4] Carattoli A, Zankari E, García-Fernández A, Voldby Larsen M, Lund O, Villa L, Møller Aarestrup F, Hasman H 2014 *In silico* detection and typing of plasmids using PlasmidFinder and plasmid multilocus sequence typing. Antimicrob Agents Chemother 58:3895–3903. doi:10.1128/AAC.02412-14. 24777092 PMC4068535

[5] Joensen KG, Tetzschner AM, Iguchi A, Aarestrup FM, Scheutz F 2015 Rapid and easy *in silico* serotyping of *Escherichia coli* isolates by use of whole-genome sequencing data. J Clin Microbiol 53:2410–2426. doi:10.1128/JCM.00008-15. 25972421 PMC4508402

[6] Larsen MV, Cosentino S, Rasmussen S, Friis C, Hasman H, Marvig RL, Jelsbak L, Sicheritz-Ponten T, Ussery DW, Aarestrup FM, Lund O 2012 Multilocus sequence typing of total genome sequenced bacteria. J Clin Microbiol 50:1355–1361. doi:10.1128/JCM.06094-11. 22238442 PMC3318499

[7] Cosentino S, Voldby Larsen M, Møller Aarestrup F, Lund O 2013 PathogenFinder-distinguishing friend from foe using bacterial whole genome sequence data. PLoS One 8:e77302. doi:10.1371/journal.pone.0077302. 24204795 PMC3810466

[8] Brouwer MS, Tagg KA, Mevius DJ, Iredell JR, Bossers A, Smith HE, Partridge SR 2015 IncI shufflons: assembly issues in the next-generation sequencing era. Plasmid 80:111–117. doi:10.1016/j.plasmid.2015.04.009. 25952328

[9] Freire MI, AbuOun M, Reichel R, La Ragione RM, Woodward MJ 2014 Sequence analysis of a CTX-M-1 IncI1 plasmid found in *Salmonella* 4,5,12:i:-, *Escherichia coli* and *Klebsiella pneumoniae* on a UK pig farm. J Antimicrob Chemother 69:2098–2101. 24729584 10.1093/jac/dku098

